# Human Embryonic Stem Cell-Derived Retinal Pigment Epithelium-Role in Dead Cell Clearance and Inflammation

**DOI:** 10.3390/ijms20040926

**Published:** 2019-02-20

**Authors:** Mária Szatmári-Tóth, Tanja Ilmarinen, Alexandra Mikhailova, Heli Skottman, Anu Kauppinen, Kai Kaarniranta, Endre Kristóf, Lyubomyr Lytvynchuk, Zoltán Veréb, László Fésüs, Goran Petrovski

**Affiliations:** 1 Department of Biochemistry and Molecular Biology, University of Debrecen, Faculty of Medicine, 4032 Debrecen, Hungary; szatmari-toth.maria@med.unideb.hu (M.S.-T.); kristof.endre@med.unideb.hu (E.K.); fesus@med.unideb.hu (L.F.); 2 Tampere University, Faculty of Medicine and Health Technology, 33014 Tampere, Finland; tanja.ilmarinen@tuni.fi (T.I.); alex.mikhailova@gmail.com (A.M.); heli.skottman@tuni.fi (H.S.); 3 School of Pharmacy, Faculty of Health Sciences, University of Eastern Finland, 70211 Kuopio, Finland; anu.kauppinen@uef.fi; 4 Department of Ophthalmology, Institute of Clinical Medicine, University of Eastern Finland, 70211 Kuopio, Finland; kai.kaarniranta@kuh.fi; 5 Department of Ophthalmology, Kuopio University Hospital, 70029 Kuopio, Finland; 6 Department of Ophthalmology, Justus-Liebig-University Giessen, Eye Clinic, University Hospital Giessen and Marburg GmbH, Campus Giessen, 35390 Giessen, Germany; Lyubomyr.Lytvynchuk@augen.med.uni-giessen.de; 7 Department of Ophthalmology, Faculty of Medicine, University of Szeged, 6720 Szeged, Hungary; vereb.zoltan@med.u-szeged.hu; 8 Center for Eye Research, Department of Ophthalmology, Oslo University Hospital and University of Oslo, Kirkeveien 166, 0450 Oslo, Norway

**Keywords:** age-related macular degeneration, anoikis, autophagy, hESC-RPE, inflammation, macrophages, phagocytosis, triamcinolone

## Abstract

Inefficient removal of dying retinal pigment epithelial (RPE) cells by professional phagocytes can result in debris formation and development of age-related macular degeneration (AMD). Chronic oxidative stress and inflammation play an important role in AMD pathogenesis. Only a few well-established in vitro phagocytosis assay models exist. We propose human embryonic stem cell-derived-RPE cells as a new model for studying RPE cell removal by professional phagocytes. The characteristics of human embryonic stem cells-derived RPE (hESC-RPE) are similar to native RPEs based on their gene and protein expression profile, integrity, and barrier properties or regarding drug transport. However, no data exist about RPE death modalities and how efficiently dying hESC-RPEs are taken upby macrophages, and whether this process triggers an inflammatory responses. This study demonstrates hESC-RPEs can be induced to undergo anoikis or autophagy-associated cell death due to extracellular matrix detachment or serum deprivation and hydrogen-peroxide co-treatment, respectively, similar to primary human RPEs. Dying hESC-RPEs are efficiently engulfed by macrophages which results in high amounts of IL-6 and IL-8 cytokine release. These findings suggest that the clearance of anoikic and autophagy-associated dying hESC-RPEs can be used as a new model for investigating AMD pathogenesis or for testing the in vivo potential of these cells in stem cell therapy.

## 1. Introduction

The retinal pigment epithelium (RPE) is a monolayer of polarized, densely pigmented cells located between the neural retina and the choriocapillaris, thus forming the outer blood-retinal barrier (BRB). The RPEs play an essential role in maintaining the homeostasis of the neural retina, including transport of nutrients and metabolites to the photoreceptors, secretion of growth factors, absorption of incident light, and phagocytosis of photoreceptor outer segments [1]. The dysfunction and death of RPE cells significantly contribute to the progression of age-related macular degeneration (AMD), which is the leading cause of blindness in the elderly of the developed world [2,3].

One of the most promising future treatment strategies for AMD is the replacement of dysfunctional RPE using cell-based transplantation therapy [4]. Human pluripotent stem cells have unlimited self-renewal characteristics [5,6] and possess the ability to differentiate into functional RPE cells [7]. The feasibility of this new approach has been studied extensively [8,9,10]. Recently, some reports on human embryonic stem cell-derived RPE (hESC-RPE) being generated on a transplantable, biopolymer coated polyimide membrane [11] as well as a parylene membrane [12] have been provided. However, the immunogenicity of hESC-RPE cells [13] has been less confirmed, and not much is known about the death and clearance modalities of such cells.

To date, several different types of cell death have been observed in the retina, such as apoptosis, anoikis, or autophagy-associated cell death, which have been implicated in the pathogenesis of AMD [14,15]. The accumulation of drusen (yellow deposits) in the space between Bruch’s membrane and the RPE layer leads to elevation of RPE cells from this membrane inducing anoikic cell death due to detachment from the extracellular matrix (ECM) [16]. On the other hand, the terms “autophagic” [17] or “autophagy-associated” cell death [18,19] or type II programmed cell death [20] have been established based on detection of increased autophagic vacuoles in the dying cells. Autophagy is present at a low or basal level in most tissues, and it is stimulated in response to stress conditions, such as insufficiency of nutrients, hypoxia, or oxidative stress [21]. The microtubule-associated protein light chain 3 (LC3) is considered to be the major marker of autophagy. The conversion of LC3 (LC3-I to LC3-II) can be followed by immunoblot analysis, the amount of the lipidated LC3-II being clearly associated with the number of autophagosomes [22]. Recently, enhanced autophagic activity was observed in the aging RPE while accumulated autophagy markers could be detected in the drusen of eyes from AMD patients [23].

The clearance of dying cells is fundamental for proper tissue homeostasis and for balance of the innate immune response [24]. Insufficient removal of dying RPE cells by non-professional phagocytes can result in debris formation and the development of dry-type of AMD [25]. Furthermore, disruption of the BRB can lead to a more advanced form of this disease, so-called wet-type of AMD, causing the appearance of professional phagocytes, such as macrophages [26,27]. The exposure of “eat-me” signals on the surface of apoptotic cells, such as phosphatidylserine, can mediate the recognition of such dying cells by phagocytes and promote their engulfment [28,29].

Triamcinolone (TC) is a conventional corticosteroid, which has anti-inflammatory and anti-angiogenic activity. The intravitreous (TC) injection is a potent therapeutic treatment for inflammatory ocular diseases including AMD [30,31,32,33]. We have previously shown that TC treatment of macrophages results in enhanced clearance of anoikic RPE cells in vitro [34]. In addition, recent observations have suggested that phagocytic uptake of autophagy-associated dying cells by macrophages triggers a pro-inflammatory response, characterized by the secretion of interleukin (IL)-6, tumor necrosis factor (TNF)-α, and IL-8 cytokines from macrophages [35].

It is widely accepted that increased low-level chronic inflammation is strongly associated with AMD; the levels of many inflammatory mediators have been found to be increased in AMD patients. These mediators are responsible for the appearance of inflammatory cells, activation of the inflammasome, promotion of neovascularization and the modulation of inflammatory processes [36].

In the current study, we aimed to establish an in vitro detection model for studying anoikic and autophagy-associated cell death in hESC-RPEs with implications to AMD. The clearance of anoikic and autophagy-associated dying RPE cells by professional phagocytes was studied using flow cytometry analysis. Additionally, the released pro-inflammatory cytokines during phagocytosis of dying hESC-RPE cells were investigated with relevance to immune tolerance, serving as a model for studying AMD in vitro and for future stem cell therapy.

## 2. Results

### 2.1. Anoikis is Induced in hESC-RPE Cells

**Figure 1:**
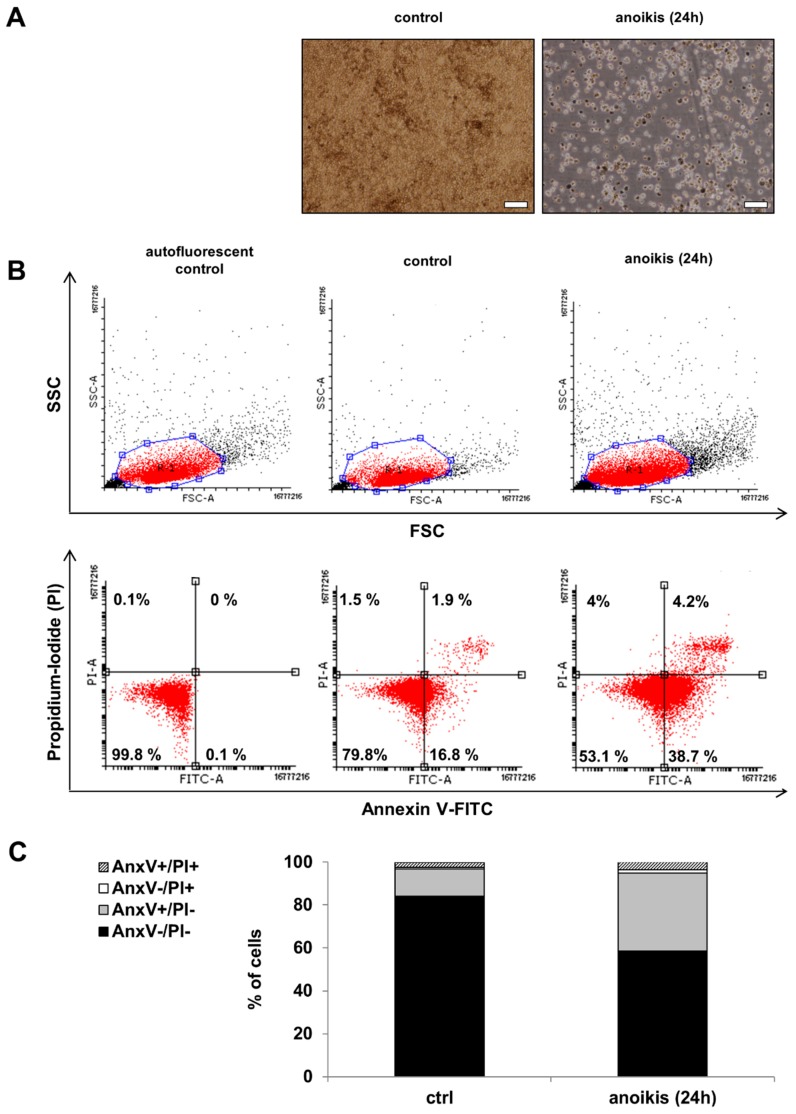
Morphological and cell death analysis after blocking the attachment of human embryonic stem cells-derived retinal pigment epithelium (hESC-RPE) cells to extracellular matrix (ECM). (**A**) Phase contrast images (10×) of untreated control hESC-RPE cells and anoikic hESC-RPE cells which were cultured on poly-2-hydroxyethylmethacrylate (poly-HEMA) coated culture dishes for 24 h to induce cell death by detachment from the extracellular matrix. Images were captured with a Nikon Eclipse TE2000-S phase contrast microscope. Scale bar indicates 20 µm. (**B**) The induction of cell death by anoikis was determined by Annexin (Anx)V-FITC/PI double staining assay. Representative dot plots of AnxV/PI measurements of anoikic dying hESC-RPE cells are shown. Top: dot plots represent the measurements of forward light scattering (FSC; X axis) vs. side light scattering (SSC; Y axis). Bottom: the horizontal axis represents the intensity of staining for Annexin V (log scale) and the vertical axis shows the intensity of staining for PI (log scale). The numbers in the quadrants indicate the percentage of different cell populations. Cells in the lower left quadrant (AnxV^−^/PI^−^) are viable, those in the lower right quadrant (AnxV^+^/PI^−^) are early apoptotic, those in the upper left (AnxV^−^/PI^+^) are necrotic and those in the upper right (AnxV^+^/PI^+^) are late apoptotic cells. Data are representative of 3 independent experiments. (**C**) The bar charts indicate the average percentage of AnxV^−^/PI^−^ (black bars), AnxV^+^/PI^−^ (grey bars), AnxV^−^/PI^+^ (white bars) and AnxV^+^/PI^+^ (striped bars) cells from 3 independent experiments.

Anoikis is a form of programmed cell death which is crucial for the maintenance of tissue homeostasis [16]. The hESC-RPE cells were plated on poly-2-hydroxyethylmethacrylate (poly-HEMA)-coated culture dishes for 24 h to induce anoikic cell death. The typical cobblestone RPE cell morphology [37,38] and high degree of pigmentation could be detected in untreated (control) hESC-RPE cells by phase-contrast microscopy. The representative phase contrast image of anoikic hESC-RPE cells shows the formation of floating aggregates (Figure 1A). The cell death rate could be assessed by Annexin V-fluorescein isothiocyanate (FITC)/propidium-iodide (PI) double staining assay using flow cytometry analysis. Representative dot plots demonstrate the gated regions based on forward light scattering (FSC; X axis), which indicates the cell size, and side light scattering (SSC; Y axis), which indicates the cell granularity (Figure 1B, top dot plots). The viable cells are both annexin-V and PI negative (lower left quadrant) and the annexin-V/PI double positive (upper right quadrant) dots indicate late apoptosis (Figure 1B, bottom dot plots). Significantly lower percentage of viable cells in anoikis-induced hESC-RPE cells compared to the control untreated cells could be detected: it decreased from 83.97 ± 4.64% to 58.5 ± 5.56%. In parallel, the ratio of only annexin V positive, early apoptotic hESC-RPE cells significantly increased from 12.9 ± 3.88% to 36.41 ± 4.15% as a result of ECM detachment. In case of anoikic cells 3.47 ± 0.66% double positive, late apoptotic cells could be detected, while the untreated controls contained only 2.38 ± 1.01% of double positive cells (Figure 1C).

### 2.2. hESC-RPE Cells Die Due to Serum Deprivation and H_2_O_2_ Co-Treatment

**Figure 2:**
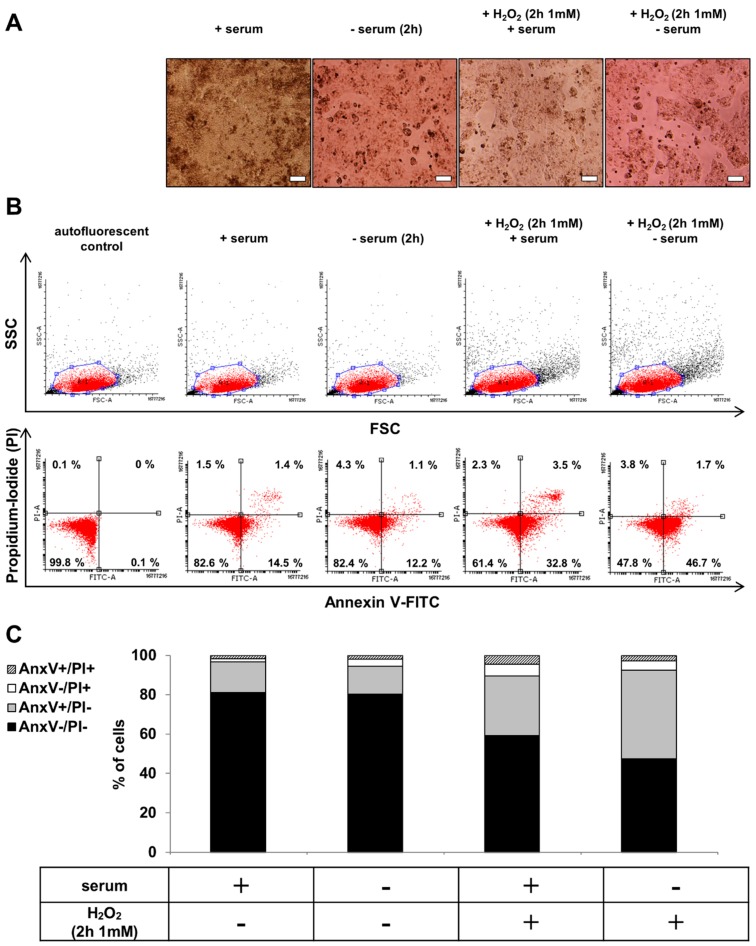
The effect of serum deprivation and H_2_O_2_ co-treatment on the morphology and cell viability of hESC-RPE cells. (**A**) Phase contrast images (10×) of untreated control, serum-deprived (2 h) and H_2_O_2_-treated (2 h, 1 mM) hESC-RPE cells in the presence or absence of serum. Images were captured with a Nikon Eclipse TE2000-S phase contrast microscope. Scale bar indicates 20 µm. (**B**) The induction of cell death by anoikis and H_2_O_2_-treatment (2 h, 1 mM) in the presence or absence of serum in hESC-RPE cells was determined by Annexin (Anx)V-FITC/PI double staining assay. Representative dot plots of AnxV/PI measurements of anoikic and H_2_O_2_-treated (2 h, 1 mM) dying hESC-RPE cells are shown. Top: dot plots represent the measurements of forward light scattering (FSC; X axis) vs. side light scattering (SSC; Y axis). Bottom: the horizontal axis represents the intensity of staining for Annexin V (log scale) and the vertical axis shows the intensity of staining for PI (log scale). The numbers in the quadrants indicate the percentage of different cell populations. Cells in the lower left quadrant (AnxV^−^/PI^−^) are viable, those in the lower right quadrant (AnxV^+^/PI^−^) are early apoptotic, those in the upper left (AnxV^−^/PI^+^) are necrotic and those in the upper right (AnxV^+^/PI^+^) are late apoptotic cells. Data are representative of 3 independent experiments. (**C**) The bar charts indicate the average percentage of AnxV^−^/PI^−^ (black bars), AnxV^+^/PI^−^(grey bars), AnxV^−^/PI^+^ (white bars) and AnxV^+^/PI^+^ (striped bars) cells from 3 independent experiments.

The untreated control hESC-RPE cells formed confluent monolayers with highly pigmented cobblestone morphology in the presence of serum replacement, while these monolayers could be disrupted as a result of serum deprivation (2 h). H_2_O_2_ treatment (2 h, 1 mM) in the presence of serum led to swelling and detachment of hESC-RPE cells, while serum deprivation and H_2_O_2_ (2 h, 1 mM) co-treatment further increased the rate of cell detachment and dead cell aggregates’ formation (Figure 2A). The cell viability was checked after serum deprivation and H_2_O_2_ co-treatment by Annexin V-FITC/PI assay using flow cytometry. Representative dot plots showed the viable (AnxV^−^/PI^−^), early apoptotic (AnxV^+^/PI^−^), necrotic (AnxV^−^/PI^+^) and late apoptotic (AnxV^+^/PI^+^) cell populations upon different conditions (Figure 2B). Serum deprivation and H_2_O_2_ co-treatment in hESC-RPE cells resulted in a significantly decreased percentage of viable cells compared to the untreated controls: it decreased from 81.19 ± 1.94% to 47.61 ± 10.63%. Simultaneously, in response to the co-treatment, significantly higher percentage of annexin V single positive hESC-RPE cells (44.91 ± 9.90%) was observed compared to untreated controls (15.68 ± 1.60%) (Figure 2C).

### 2.3. Autophagy is Induced in hESC-RPE Cells Treated by Serum Deprivation and H_2_O_2_ Co-Treatment

**Figure 3:**
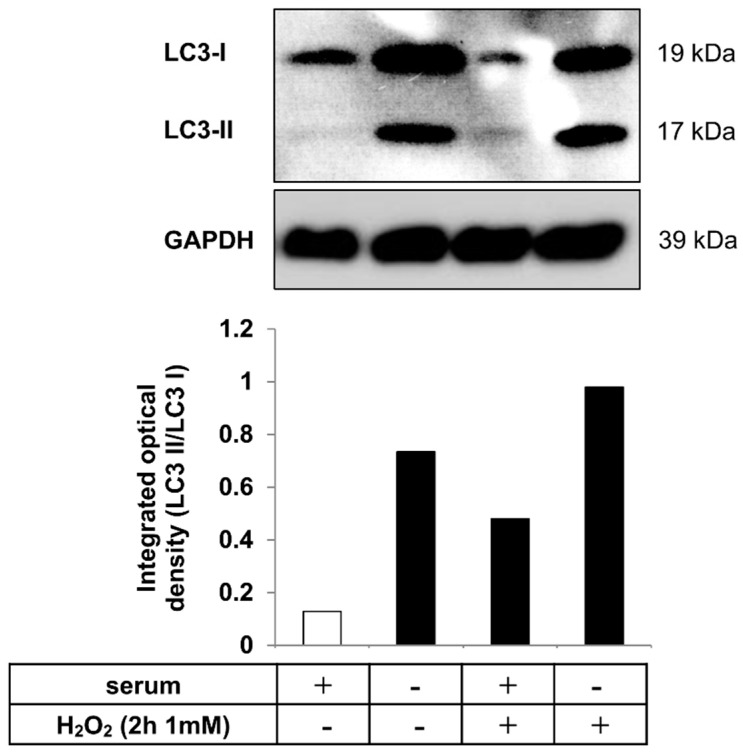
Autophagy induction as a result of serum deprivation and H_2_O_2_ co-treatment in hESC-RPE cells. Representative western blot image for the expression of LC3 in hESC-RPE cells treated with 1 mM H_2_O_2_ for 2 h in the presence or absence of serum. Integrated optical density was determined by densitometry for quantification of the LC3-II/LC3-I ratio using the ImageJ software. GAPDH was used as a loading control. Data are representative of three independent experiments.

Autophagy is a highly regulated process, which can maintain homeostasis by protein degradation and turnover of damaged or unnecessary organelles during new cell formation [39,40,41]. Under different conditions, autophagy can act to promote cell death through an autophagy-associated process which is distinct from apoptosis; it depends on the level of autophagy activation [21,42,43]. The most extensively studied stimuli that induce autophagy are oxidative stress [44,45], starvation or serum-deprivation [46,47]. Detection of increased autophagic markers in dying cells, such as microtubule-associated protein 1 light chain 3 (LC3), serve as an indicator of autophagy-associated cell death. During the process of autophagy, the cytosolic form of LC3 (LC3-I) is converted to the lipidated, autophagosome-membrane-bound form (LC3-II) [22]. Serum deprivation and H_2_O_2_ (2 h, 1 mM) co-treatment was used as an autophagy inducer in hESC-RPE cells. The level of LC3 protein was analyzed by Western blot and the LC3-II/LC3-I ratio was quantified by densitometry. An increased LC3-II/LC3-I ratio could be detected upon such co-treatment compared to untreated control. These data suggest that serum deprivation and H_2_O_2_ co-treatment results in induction of autophagy (Figure 3).

### 2.4. Macrophages can Efficiently Engulf Anokic and Autophagy-Associated Dying hESC-RPE Cells in vitro

**Figure 4:**
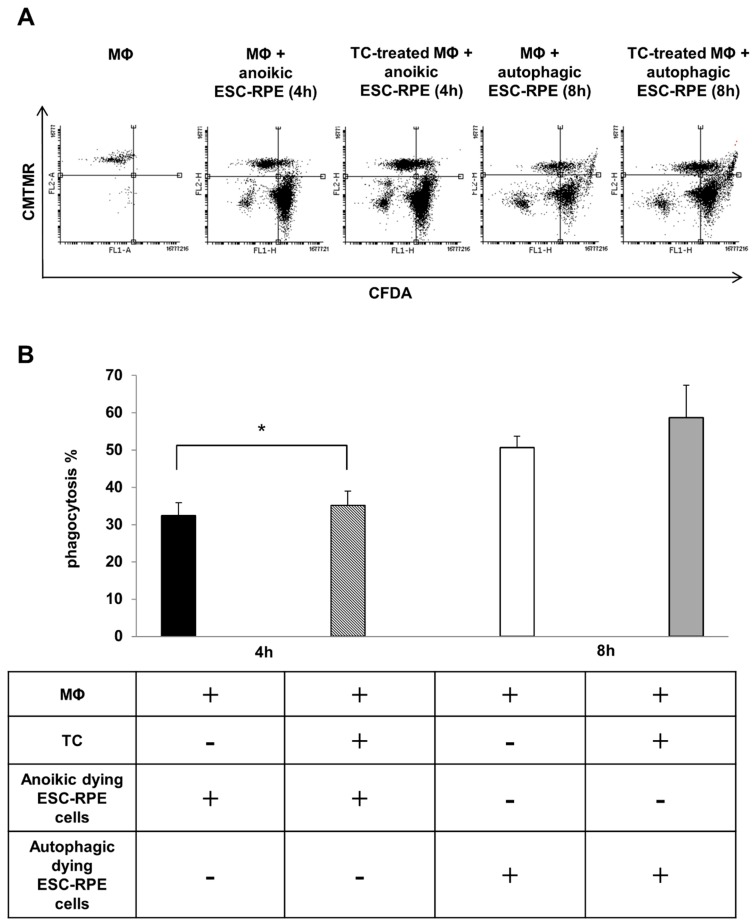
The clearance of anoikic and autophagy-associated dying hESC-RPE cells by macrophages. (**A**) Representative flow cytometry dot plots demonstrating phagocytosis of anoikic and autophagy-associated dying hESC-RPE cells by macrophages after 4 h and 8 h co-incubation, respectively. Macrophages were pre-treated with 1 µM triamcinolone (TC) for 48 h. The horizontal axis represents the intensity of staining for CFDA (log scale) and the vertical axis shows the intensity of staining for CMTMR (log scale). Cells in the upper right quadrant indicate the engulfed hESC-RPE (CFDA-labeled) cells by macrophages (CMTMR-labeled). Data are representative of 3 independent experiments. (**B**) The phagocytosis rate of anoikic and autophagy-associated dying hESC-RPE cells by untreated and TC-pre-treated (48 h, 1 μM) macrophages after 4 h and 8 h co-incubation, respectively, is shown as determined by flow cytometry analysis. Bars represent the mean ± SD of 3 independent experiments, * *p* < 0.05.

The phagocytosis of anoikic and autophagy-associated dying hESC-RPE cells by macrophages was analyzed by flow cytometry after 4 h and 8 h of co-incubations, respectively. Furthermore, the effect of TC treatment (48 h, 1 μM) on the phagocytosis capacity of professional phagocytes was also examined (Figure 4A). The anoikic hESC-RPEs were efficiently removed by macrophages, the average phagocytosis being 32.40 ± 3.45% at 4 h of co-incubation. A more efficient phagocytosis rate was found when autophagy-associated dying hESC-RPE cells were engulfed by macrophages over 8 h of co-incubation: 50.72 ± 2.98%. TC treatment moderately, yet significantly enhanced the engulfing capacity of macrophages for anoikic dying cells (35.15 ± 3.85%), which was similar, yet not significantly increased in case of engulfing autophagy-associated dying hESC-RPEs (Figure 4B).

### 2.5. The Phagocytosis of Anoikic and Autophagy-Associated Dying hESC-RPE Cells by Macrophages Induces Release of Pro-Inflammatory Cytokines

**Figure 5:**
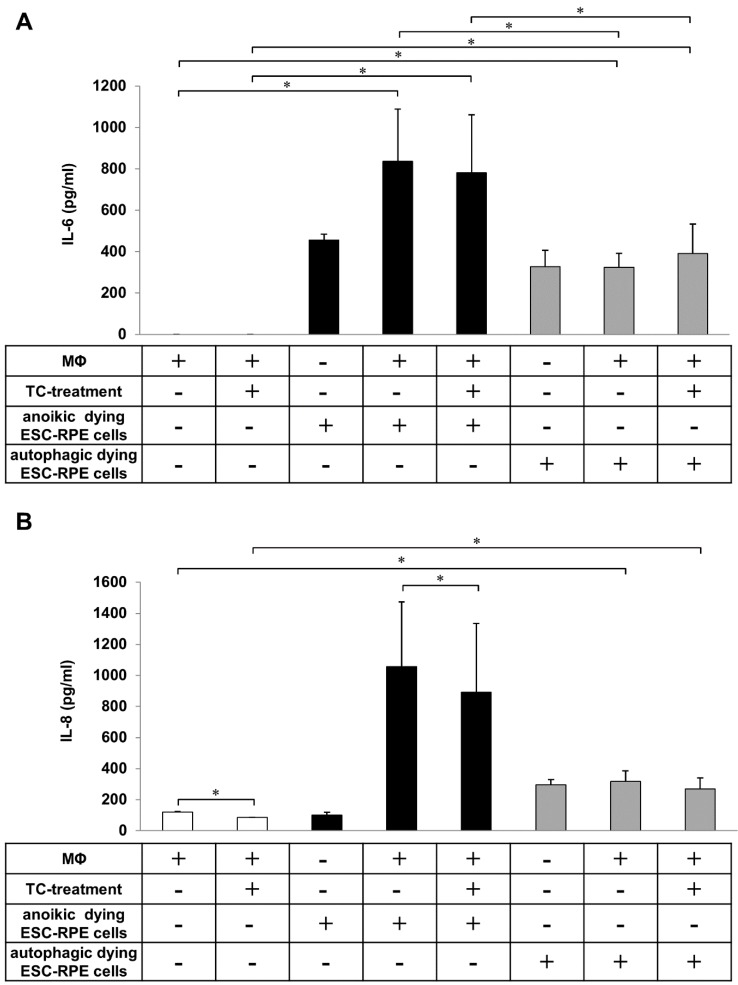
Determination of IL-6 and IL-8 release during the engulfment of anoikic and autophagy-associated dying hESC-RPE cells by macrophages. Anoikic dying hESC-RPE cells (left panels) and autophagy-associated dying hESC-RPE cells (right panels) were co-incubated with untreated and triamcinolone (TC)-treated (48 h, 1 μM) macrophages for 4 h and 8 h, respectively, then the supernatants were collected, and the level of secreted IL-6 (**A**) and IL-8 (**B**) cytokines were measured by ELISA. Bars represent the mean ± SD of 3 independent experiments, * *p* < 0.05.

The induction of inflammatory responses in macrophages during engulfment of apoptotic and necrotic cells has been well described [28,48,49,50,51]. However, to date, only a few studies have investigated the inflammatory effect of clearance of anoikic and autophagy-associated dying cells [52]. Therefore, the release of pro-inflammatory cytokines by macrophages as a result of uptake of anoikic and autophagy-associated dying hESC-RPE cells in vitro was examined. Anoikic and autophagy-associated dying hESC-RPE cells induced by H_2_O_2_ (2 h, 1 mM) were co-incubated with macrophages for 4 h and 8 h, respectively, and the cell culture supernatants were collected for cytokine release study. In parallel, the anti-inflammatory effect of the glucocorticoid TC (48 h, 1 μM) on the secretion of IL-6 and IL-8 cytokines during phagocytosis of dying cells by macrophages was monitored (Figure 5). No IL-6 secretion by macrophages could be detected when no interaction with the dying cells occurred (control state). The clearance of anoikic hESC-RPE cells by macrophages resulted in a robust and significant increase in IL-6 secretion (836.33 ± 252.27 pg/mL), which decreased upon TC treatment (780.87 ± 279.18 pg/mL) (Figure 5A). Significantly lower levels of IL-6 release were detected during autophagy-associated dying cells’ uptake (324.37 ± 67.43 pg/mL). Similar secretion pattern for IL-8 was found in comparison to the low amount of IL-8 secreted by TC-treated (120.92 ± 1.90 pg/mL) and untreated (84.40 ± 2.48 pg/mL) macrophages (in absence of dying cells). Interestingly, the engulfment of anoikic cells induced a high increase in IL-8 production (1057.33 ± 416.56 pg/mL) by macrophages, the level of which significantly decreased upon TC-treatment (892.11 ± 442.08 pg/mL). Lower secretion of IL-8 was detected during the clearance of autophagy-associated dying cells (318.13 ± 67.99 pg/mL) compared to anoikic ones, yet this release was significant compared to the background secretion by macrophages alone or in the presence of TC (Figure 5B). TC treatment caused no significant differences in the secretion of IL-6 and IL-8 during phagocytosis of autophagy-associated dying cells by macrophages.

## 3. Discussion

To date, specific therapy to treat AMD is not available, probably due to the complex multifactorial nature of this disease. Therefore, the establishment of novel models for studying the pathogenesis of AMD which can help generate new therapeutic approaches is much needed. In the recent years, hESC technologies have progressed rapidly, with several groups having described successful RPE differentiation strategies from hESCs and induced pluripotent stem cells [53,54,55,56]. This has certainly offered a possibility to understand the mechanisms of AMD disease more accurately.

We have previously demonstrated that hESC-RPE cells formed highly polarized, hexagonal, cobblestone-like morphology with tight epithelial structure and high pigmentation rate in vitro. It was demonstrated that such cells express RPE cell-specific markers such as *MITF*, *RPE65*, *BEST1*, *OTX2v1*, *PMEL*, *PEDF*, *TYR* at gene level, and MITF, CRALBP, RPE65, and ZO-1 at protein level. Furthermore, the integrity and barrier characteristics of these cells was described [57,58,59] as well as their transport of different types of drugs [60]. The hESC derived RPE-like cells showed very similar functional properties as the native RPE. Taken together, these data suggest that hESC-RPEs can serve as a relevant in vitro model to study the mechanisms of RPE-associated diseases, including retinal degenerations. To the best of our knowledge, neither the death modalities nor the clearance of dying hESC-RPEs by professional phagocytes have been studied so far and no data exist whether they trigger any release of inflammatory cytokines by innate immune cells.

In the pathogenesis of AMD, the accumulation of drusen deposits between the Bruch’s membrane and the RPE cell layer can induce anoikis as a result of RPE detachment from the ECM [16,61,62]. Our research group recently published a novel in vitro model for the induction of anoikic cell death in ARPE-19 cell line and in primary hRPE cells, using poly-HEMA-covered dishes over a 24 h cultivation, thus blocking cell attachment to the culture plate [63]. The present study followed the same protocol for anoikis induction and analysis in hESC-RPE cells [64,65,66,67]. The FITC-labeled Annexin V (AnxV) is a widely used PS-binding protein for the detection of apoptotic cells by flow cytometry (early apoptosis can be depicted as AnxV^+^/PI^−^ population) [68,69]. PI cannot pass through the intact membranes of viable cells, but when this membrane in dead cells is disturbed, PI can leak into the cells and bind to nucleic acids. It is used to detect necrotic cell death by flow cytometry (AnxV^−^/PI^+^ population) [70]. Our anoikic hESC-RPE cells showed high rate of AnxV positivity (36%), similar to the rate observed in ARPE-19 (19%) [63].

The retina is constantly subjected to oxidative stress as a result of high oxygen consumption, constant light exposure and high mitochondrial activity, which leads to the release of reactive oxygen species (ROS) in the RPE [71]. As a defense response reaction against the oxidative stress, production of antioxidants increases. However, the expression of anti-oxidative enzymes in RPE cells decreases with age, which can be the cause of serious oxidative damage of the retina and ultimately progression of AMD [72,73,74]. H_2_O_2_ is one of the major ROS which is naturally produced in human eyes in vivo and its level increases under pathological conditions [75]. H_2_O_2_-exposure is a well-known in vitro model for triggering oxidative stress [76,77] and increasing evidence suggests a relation with high autophagic activity [78,79,80]. Healthy cells are characterized by having a basal level of autophagy [21]. Several studies report that autophagy is implicated in the pathogenesis of AMD [15,23,81]. Furthermore, the accumulation of increased autophagy markers has been shown as a result of H_2_O_2_-treatment in RPE cells [82,83,84]. Recently, we described the induction of autophagy-associated cell death in ARPE-19 and primary hRPE cells by serum deprivation and H_2_O_2_ (2 h, 1 mM) [85]. The phenomenon means an induction of high level of autophagy first, which is the cause of cell death, thus death by autophagy occurs; in contrast, another process is referred to as death with autophagy, in which cell death is induced first which is the cause of autophagy induction—the latter being a consequence of cell death. Same concentration and time exposure of H_2_O_2_-treatment for induction of autophagy-associated cell death in hESC-RPE cells was used here: approximately 40% of hESC-RPE cells were single positive for AnxV, which was similar to the results obtained in ARPE-19 cells; moreover, increased LC3-II/LC3-I ratio was observed by Western blot analysis.

The retina represents an “immunologically privileged” tissue, which is maintained by several mechanisms, such as the existence of BRB to prevent certain substances from entering the retina, or the inhibitory microenvironment with immunosuppressive factors serving as inhibitors of the activity of immune-competent cells. In addition, the retina has an essential role in the regulation of systemic immune responses [86]. In the case of neovascularized form of AMD, the breakdown of BRB can lead to highly immunogenic environment, and recruitment and activation of macrophages [87,88]. The clearance of damaged cellular components and dying cells is a crucial function of macrophages for visual homeostasis [89]. Apoptotic cells expose “eat me” signals on their surface to trigger their own recognition and engulfment by macrophages [90,91]. To date, several “eat me” markers have been identified; among them, PS has been the most studied and well-accepted [92,93]. Phagocytes express specific cell surface receptors to recognize PS, including Mer tyrosine kinase (Mertk), which is a member of the TAM subfamily [94,95]. In this process, growth arrest-specific protein 6 (Gas6) molecules serve as a bridging molecule by binding to Mertk receptors on macrophages and PS on apoptotic cells [96,97]. Our previous studies demonstrated the clearance dynamics of anoikis and autophagy-associated dying ARPE-19 and primary hRPE cells by macrophages; moreover, the enhancing effect of TC treatment in the phagocytic capacity could be shown [63]. In addition, we recently reported a key role of Mertk receptors in the regulation of TC-enhanced clearance of anoikic dying RPE cells by macrophages and the enhancing effect of Gas6 on their phagocytic capacity [34]. In the present study, the phagocytosis of dying hESC-RPE by anoikis or autophagy-associated process were being studied. Compared to our previous results, dying hESC-RPE cells were removed by macrophages much more efficiently than dying ARPE-19 and hRPE cells. In addition, macrophages engulfed higher amounts of dying hESC-RPE cells as a result of TC-pre-treatment.

The inflammatory response of macrophages during apoptotic and necrotic cell removal has been well described [28,48,49,50], but less data is available regarding the immunogenic features of phagocytes upon engulfment of anoikic or autophagy-associated dying cells. It is a widely accepted dogma that the uptake of apoptotic cells is an immunologically silent process, accompanied by the release of anti-inflammatory cytokines such as IL-10 or TGF-β by the phagocytes [98,99,100,101]. By contrast, the phagocytosis of necrotic cells by macrophages usually results in a pro-inflammatory and immunostimulatory effect [102,103,104]. However, if the apoptotic cells are not properly removed, the remained cells begin to lose their membrane integrity, which eventually leads to progression into secondary necrosis, as well as induction of inflammatory response [105,106,107]. We have previously shown that engulfment of autophagic dying MCF-7 cells causes a pro-inflammatory response in macrophages with enhanced secretion of IL-6, TNF-α and IL-8 cytokines [52]. In line with this, we could recently demonstrate a similar effect in case of engulfment of autophagy-associated dying ARPE-19 and hRPE cells [85].

In the present study, a high amount of IL-6 and IL-8 production during phagocytosis of anoikic dying hESC-RPE cells was observed. The improper clearance of anoikic dying cells might contribute to trigger the release of pro-inflammatory mediators. In addition, a small ratio of secondary necrotic population as a result of cell culturing on poly-HEMA-covered dishes over a 24 h period cannot be excluded as a contributing factor, which possibly can occur in vivo during long-standing neovascular type of AMD. Furthermore, a decreased release of IL-6 and IL-8 production during the engulfment of anoikic dying hESC-RPE cells was found upon the anti-inflammatory effect of the glucocorticoid TC. In accordance with our previous results [52], we could show that hESC-RPE cells dying through autophagy-associated process can induce a pro-inflammatory response in human macrophages. On the other hand, it has been reported that hESC-RPE cells can decrease the T lymphocyte activation [108].

Overall, these findings suggest that the clearance of anoikic and autophagy-associated dying hESC-RPE cells can be used as a new model for investigating AMD pathogenesis or for testing the in vivo potential or immunological consequence of using such cells for stem cell therapy.

## 4. Materials and Methods

### 4.1. Ethic Statement

Human embryonic stem cell (hESC) lines were established from surplus or low quality preimplantation embryos, which could not be used for in vitro fertilization treatment, under the auspices of the approval of the National Authority for Medicolegal Affairs Finland (identification code 1426/32/300/05, 19.05.2005). The derivation, characterization, and differentiation of hESC lines were approved by the Ethics Committee of Pirkanmaa Hospital District (identification code R05116, 16.12.2016).

Buffy coats were provided anonymously by the Finnish Red Cross Blood Service where blood was taken from healthy volunteers. For these studies approval was obtained from the ethics committee of Kuopio University Hospital (42/2014).

### 4.2. Human ESC Culture, RPE Differentiation, and Treatment

The Regea08/017 (46, XX) pluripotent hESC line was derived from blastocyst stage embryos, as published by Skottman [109]. Both hESC lines were maintained on inactivated human foreskin fibroblast feeder cells (hFF) (CRL-2429, ATCC, Manassas, VA, USA), cultured at 37 °C, 5% CO_2_ in KnockOut™ Dulbecco’s Modified Eagle Medium (KO-DMEM, cat. 10829018) supplemented with 20% KnockOut^TM^ Serum Replacement (KO-SR, cat. 10828028), 2 mM Glutamax (cat. 35050038), 0.1 mM 2-mercaptoethanol (cat. 31350010) (all from Thermo Fisher Scientific, Waltham, MA, USA), 1% Minimum Essential Medium non-essential amino acids (cat. 13-114E), 50 U/mL Penicillin/Streptomycin (cat. 17-602E) (both from Lonza, Basel, Switzerland), and 8 ng/mL human basic fibroblast growth factor (bFGF, cat. 100-18B) (Pepro-Tech, NJ, USA). Fresh culture medium was changed 3 times a week. The differentiation and enrichment process of RPE from pluripotent hESCs was carried out as previously described [58,110] Cell growth and pigmentation were followed weekly by Nikon Eclipse TE2000-S phase contrast microscope (Nikon Instruments Europe B.V. Amstelveen, The Netherlands). Prior cell death experiments, the confluent hESC-RPE cell cultures were matured on CIV-coated (5 µg/cm^2^) well plates for 6 weeks in RPE basic medium [58], after which the cells were detached from the cell culture plates using trypsin-EDTA (Sigma-Aldrich, St. Louis, MI, USA, T3924). For the induction of autophagy-associated cell death, cells were plated over a 24 h period and co-treated by serum deprivation and hydrogen-peroxide (H_2_O_2_) (2 h, 1 mM) (Sigma-Aldrich, H1009). Anoikic cell death was induced by detachment from the extracellular matrix (anoikis), when hESC-RPE cells were plated on poly-2-hydroxyethyl methacrylate (poly-HEMA) (Sigma-Aldrich, 529265) coated culture dishes for 24 h.

### 4.3. Assays of Cell Death

Anoikic cell death, serum deprivation and H_2_O_2_ (2 h, 1 mM) co-treatment induced cell death was determined by the Annexin-V-fluorescein isothio-cyanate Apoptosis Detection Kit (MBL, Woburn, MA, USA, BV-K-101-4) according to the manufacturer’s recommendations. Annexin V-FITC staining is based on the detection that phosphatidylserine (PS) translocates from the intracellular plasma membrane to the cell surface during apoptosis and specifically binding fluorochrome-labeled Annexin V. In parallel, the observation that Propidium Iodide (PI) permeates through the disturbed cell membranes and binds to DNA can be used to assess necrotic cell death. The percentage of Annexin-V and/or PI positive cells was analyzed by BD Accuri C6 flow cytometer (BD Biosciences, San Jose, CA, USA) [111,112].

### 4.4. Antibodies and Immunoblotting

Cells were harvested and washed with PBS (phosphate-buffered saline) (HyClone, Logan, UT, USA, SH30028.02), suspended in M-PER (mammalian protein extraction reagent) cell lysis reagent (Thermo Fisher Scientific, 78501) and then the insoluble cellular material was removed by centrifugation. The protein concentration was determined by using Bradford reagent (Sigma-Aldrich, B6916) and the lysates were mixed with 5× Laemmli loading buffer, boiled for 10 min. Equal amounts of protein (20 µg) were separated on 15% SDS- polyacrylamide gel, and transferred onto a PVDF Immobilon-P Transfer Membrane (Merck-Millipore, Darmstadt, Germany, IPVH00010; pore size 0.45 µm). The membranes were blocked in Tris-buffered saline containing 0.05% Tween-20 (Sigma-Aldrich, P-1379) (TBS-T) and 5% nonfat milk (AppliChem, Darmstadt, Germany, A0830) for 1 h. To detect autophagy induction, anti-LC3 rat polyclonal antibody (Novus Biologicals, Littleton, CO, USA, NB100-2220) was used which recognizes both LC3-I and LC3-II. Then membranes were immunoblotted overnight at 4 °C with primary antibody against LC3 (1:2000) in TBS-T containing 1% nonfat milk, followed by incubation with horseradish-peroxidase (HRP)-conjugated anti-rat secondary antibody (Sigma-Aldrich, A9169) (1:10000) for 1h at room temperature. Then membrane was probed for 1 h at room temperature with GAPDH antibody (1:5000) (Covalab, Villeurbanne, France, mab50114) in TBS-T containing 1% nonfat skimmed milk, followed by incubation with HRP-conjugated species anti-mouse secondary antibody (1:10000) (Sigma-Aldrich, A5906) for 1h at room temperature. GAPDH antibody was used as a loading control. Immunoreactive products were visualized using Immobilon Western chemiluminescence substrate (Merck-Millipore, WBLLS0500). Densitometry was carried out using the Image J software.

### 4.5. Phagocytosis Assay

Human monocytes were isolated from ‘buffy coats’ of healthy blood donors by density gradient centrifugation using Ficoll–Paque Plus (Amersham Biosciences, NJ, USA, 17-1440-02). CD14^+^ cells were separated by magnetic sorting with MACS using CD14 human MicroBeads (Miltenyi Biotec, BergischGladbach, Germany, 130050201). Human macrophages were cultured through a 5-day differentiation using 5 ng/mL macrophage colony-stimulating factor (M-CSF) (Peprotech EC, London, UK, PPT-300-25-B) at 37 °C in Iscove’s Modified Dulbecco’s Medium (IMDM) (Gibco, Paisley, UK, 21056-023) supplemented with 10% human AB serum (Sigma-Aldrich, H4522) and 10000 U/mL penicillin- 10 mg/mL streptomycin (Sigma-Aldrich, P4333) [34].

Phagocytes were pre-treated with 1 µM triamcinolone (TC) (Sigma-Aldrich, T6501), 48 h prior to the assay, and were labelled with CMTMR (5-(and-6)-(((4-chloromethyl)benzoyl) amino)tetramethylrhodamine) (Molecular Probes, Eugene, OR, USA, C2927) (16 h, 7.5 µM), as described previously [34,63]. CFDA-SE-stained (carboxyfluores-ceindiacetate-succinimidyl ester) (Molecular Probes, C1157) (2 h, 12.5 µM) anoikic and H_2_O_2_-induced (2 h, 1 mM) autophagy-associated dying hESC-RPE cells were engulfed by macrophages. Before the phagocytosis assay, the labelled macrophages and dying cells were washed twice with PBS. Phagocytes were co-cultured with anoikic and autophagy-associated dying hESC-RPE cells for 4 h and 8 h, respectively, at a ratio of 1:3 at 37 °C, 5% CO_2_ atmosphere in IMDM medium in the absence of human 10% AB serum. The whole-cell mixture was collected by trypsinization to remove bound but not engulfed dying cells. Then cells were centrifuged, washed twice in PBS and fixed in 1% PBS-buffered paraformaldehyde (pH 7.4). The clearance of anoikic and autophagy-associated dying cells by macrophages was quantified using flow cytometry. Phagocytosis rate was determined as percent phagocytic cells (CMTMR positive) that have engulfed dying cells (positive for both CMTMR and CFDA-SE) [111].

### 4.6. Quantification of IL-6 and IL-8 Release by ELISA

The released pro-inflammatory cytokines by the macrophages was investigated during phagocytosis of dying hESC-RPE cells. Differentiated macrophages were co-cultured with anoikic dying and H_2_O_2_-treated (2 h, 1 mM) hESC-RPE cells for 4 h and 8 h, respectively, and culture supernatants were harvested and stored for cytokine measurements. Macrophages were either treated with 1 μM TC for 48 h or left untreated prior to starting the phagocytosis assays. The concentration of IL-6 (pg/mL) and IL-8 (pg/mL) cytokines was measured from the collected cell culture media using Human IL-6 ELISA OptEIA^TM^ BD Biosciences, 555220) and Human IL-8 ELISA OptEIA^TM^ kits (BD Biosciences, 55524) according to the manufacturer’s specifications.

### 4.7. Statistical Analysis

Results are expressed as the mean ± SD or mean ± SEM for the number of assays indicated. For comparison of two groups Student’s *t*-test was used. Values of *p* < 0.05 were considered statistically significant.

## Data Availability

All data in the manuscript will be publicly made available upon acceptance.

## Abbreviations

2-MEA	2-mercaptoethanol
5(6)-CFDA-SE	5-(and-6)-carboxyfluorescein diacetate, succinimidyl ester
CMTMR	5-(and-6)-(((4-chloromethyl)benzoyl)amino)tetramethylrhodamine
AMD	age-related macular degeneration
AV	autophagic vacuole
CNV	choroideal neovascularization
DMEM	Dulbecco’s modified Eagle’s medium
EDTA	ethylenediaminetetraacetic acid
FCS	fetal calf serum
FITC	fluorescein isothio-cyanate
FACS	fluorescence-activated cell sorter
HRP	horseradish-peroxidase
hRPE	human retinal pigment epithelium
H_2_O_2_	hydrogen peroxide
IL	interleukin
IMDM	Iscove’s Modified Dulbecco’s Medium
LC3	light-chain-3
MCSF	macrophage colony stimulating factor
PFA	paraformaldehyde
PBS	phosphate-buffered saline
PS	phosphatidyl serine
poly-HEMA	poly(2-hydroxyethylmethacrylate)
PI	propidium iodide
SD	standard deviation
TC	triamcinolone
TBS-T	tris buffered saline containing 0.05 % tween-20
